# Thiostrepton: A Novel Therapeutic Drug Candidate for *Mycobacterium abscessus* Infection

**DOI:** 10.3390/molecules24244511

**Published:** 2019-12-10

**Authors:** Tae Ho Kim, Bui Thi Bich Hanh, Guehye Kim, Da-Gyum Lee, June-Woo Park, So Eui Lee, Jae-Sung Kim, Byoung Soo Kim, Sungweon Ryoo, Eun-Kyeong Jo, Jichan Jang

**Affiliations:** 1 Molecular Mechanisms of Antibiotics, Division of Life Science, Research Institute of Life Science, Gyeongsang National University, Jinju 52828, Korea; 2 Division of Applied Life Science (BK21plus Program), Gyeongsang National University, Jinju 52828, Korea; 3 Clinical Research Centre, Masan National Tuberculosis Hospital, Changwon 51755, Korea; 4 Future Environmental Research Center, Korea Institute of Toxicology, Jinju 52834, Korea; 5 Human and Environmental Toxicology Program, Korea University of Science and Technology (UST), Daejeon 34113, Korea; 6 Department of Microbiology, College of Medicine, Chungnam National University, Daejeon 35015, Koreahayoungj@cnu.ac.kr (E.-K.J.); 7 Infection Control Convergence Research Center, Chungnam National University, Daejeon 35015, Korea; 8 Department of Bionano Technology, Hanyang University, Seoul 04763, Korea; 9 Department of Radiopharmaceutical Research, Korea Institute of Radiological and Medical Sciences, Seoul 01812, Korea

**Keywords:** thiostrepton, *Mycobacterium abscessus*, zebrafish bacterial infection, drug resistance, non-tuberculous mycobacteria

## Abstract

*Mycobacterium abscessus* is a rapid-growing, multidrug-resistant, non-tuberculous mycobacterial species responsible for a variety of human infections, such as cutaneous and pulmonary infections. *M. abscessus* infections are very difficult to eradicate due to the natural and acquired multidrug resistance profiles of *M. abscessus*. Thus, there is an urgent need for the development of effective drugs or regimens against *M. abscessus* infections. Here, we report the activity of a US Food and Drug Administration approved drug, thiostrepton, against *M. abscessus*. We found that thiostrepton significantly inhibited the growth of *M. abscessus* wild-type strains, subspecies, clinical isolates, and drug-resistant mutants in vitro and in macrophages. In addition, treatment of macrophages with thiostrepton significantly decreased proinflammatory cytokine production in a dose-dependent manner, suggesting an inhibitory effect of thiostrepton on inflammation induced during *M. abscessus* infection. We further showed that thiostrepton exhibits antimicrobial effects in vivo using a zebrafish model of *M. abscessus* infection.

## 1. Introduction

*Mycobacterium abscessus* (*Mab*) complex (MABSC) is the most common rapidly growing mycobacterium that causes lung and skin diseases in humans. Based on genome analysis, MABSC is sub-classified into three different subspecies, namely *Mab abscessus*, *Mab bolletii*, and *Mab massiliense* [1]. This species has been isolated from soft skin tissues following infection due to surgery, cosmetic procedures, acupuncture, and body piercing, as well as from hosts with pulmonary diseases, especially cystic fibrosis or bronchiectasis [1]. *Mab* and MAC (*Mycobacterium avium*, *Mycobacterium intracellulare*, and *Mycobacterium chimaera*) are the most frequent pathogens associated with pulmonary diseases, accounting for >90% of the total cases reported [2]. They are also known as nontuberculous mycobacteria (NTM) because they do not cause tuberculosis. Although MAC is the most common NTM clinical pulmonary isolate in the United States followed by MABSC, in some parts of Asia, MABSC is the predominant pulmonary NTM pathogen [3]. For example, pulmonary *M. abscessus* is the most common NTM isolated from Singapore and Okinawa, which is located in the southernmost region of Japan, although there have been geographic differences [4,5].

According to the American Thoracic Society/Infectious Diseases Society of America (ATS/IDSA), the recommended *Mab* treatment includes multidrug therapy using macrolides (clarithromycin, CLA and amikacin, AMK), as well as beta-lactams (imipenem, IMP and cefoxitin, CFX) [6,7]. Clinical experts recommend a combination of AMK, CLA, and CFX for 1–2 months, followed by fluoroquinolone to treat *Mab* pulmonary disease. However, the use of this regimen cures only 50% of all patients, most of whom relapse or die [8]. This poor success rate is due to rapidly emerging drug resistance, side effects, or toxicity in the majority of patients [9]. Among them, CLA is known to be the main drug used in the treatment of *Mab* [10]. However, *Mab* infections are very difficult to eradicate; these bacteria are one of the most drug-resistant mycobacteria because of their natural and acquired multidrug resistance profiles [11,12]. Even representative anti-tuberculosis drugs, such as isoniazid and rifampicin, lose their activity against *Mab* due to presumably the efflux pump mechanism and inactivation by ADP-ribosyltransferase, respectively [13,14]. These poor treatment outcomes are complicated by the emergence of strains displaying inducible resistance against macrolides, such as azithromycin and CLA [15]. Inducible resistance to macrolides is mediated by the *erm*(41) gene and its gene product, erythromycin ribosome methyltransferase, that modifies the ribosomal binding site of CLA [16]. Furthermore, some screens for compounds active against *M. abscessus* showed difficulties in the identification of druggable compounds due to low hit rates compared to those for other mycobacterial species, such as *M. tuberculosis*. For instance, although Malin et al. conducted a whole-cell phenotypic screen using 10,000 synthetic small molecules, this screen generated only seven hits, which is 10 times lower than the hits obtained for *M. tuberculosis* [17]. Therefore, a new drug candidate that can effectively treat *Mab* infection is urgently needed.

Thiostrepton (TST) is a quinaldic acid moiety containing a natural thiopeptide, and is an FDA- approved antimicrobial drug for animal use [18,19]. TST is one of the most effective translational blockers and the mechanism of action of TST is unique compared to the other current drugs that target bacterial ribosome, such as erythromycin. The mechanism of action of TST involves binding to nucleotides A1065 and A1095 on helices 43 and 44 of 23S rRNA as well as proline residues within the N-terminal domain of ribosomal protein, uL11 [19,20]. Although the initial use of TST was as a topical veterinary antibiotic, TST mediates effective growth inhibition against methicillin-resistant *Staphylococcus aureus* (MRSA), methicillin-resistant *Enterococcus faecium*, penicillin-resistant *Streptococcus pneumoniae*, and vancomycin-resistant enterococci [21]. In addition, as one of the 1514 compounds screened to select an effective compound against *Mycobacterium tuberculosis*, TST exhibited promising activity [22]. In a study with *Mycobacterium marinum*, which is a mycobacterial species most closely related to members of the *M. tuberculosis* complex, TST induced endoplasmic reticulum (ER) stress-mediated autophagy to activate host cell defense [19]. Furthermore, TST acts as a potent anti-cancer agent against breast cancer cells with minimal toxicity against non-cancer cells [23,24].

Here, we describe the effects of TST against *Mab*. TST was effective against all subspecies of the MABSC, clinical isolates, including AMK-, CFX-, or CLA-resistant mutants. This activity was also confirmed in vitro, and in the zebrafish (ZF) model of infection.

## 2. Results

### 2.1. TST Inhibits In Vitro Growth of Mab CIP 104,536 and MABSC

**Figure 1:**
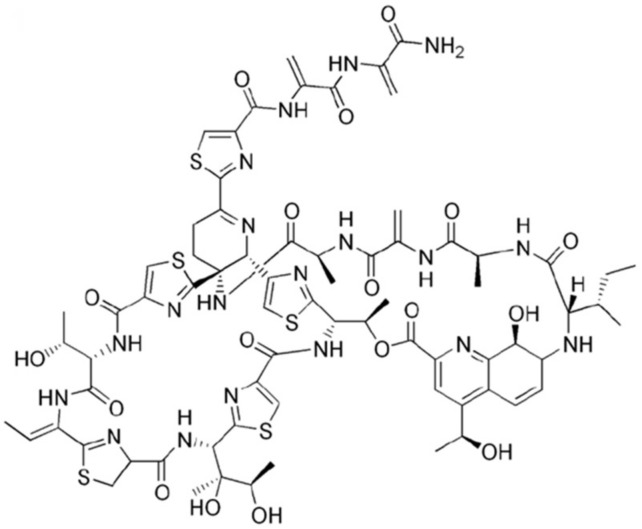
Chemical structure of Thiostrepton (TST).

**Figure 2:**
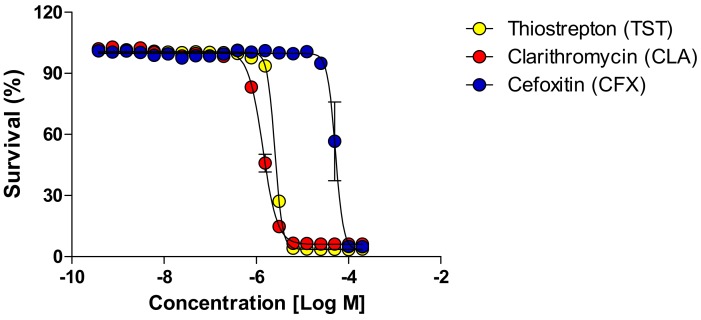
In vitro activity of TST. The activity of TST against *Mab abscessus* CIP 104,536 S morphotype in comparison with CLA and CFX in cation-adjusted Mueller–Hinton medium.

To investigate whether TST (Figure 1) exhibits a growth-inhibitory effect on *Mab*, we conducted a drug susceptibility test for the two different *Mab* CIP 104,536 morphortypes in cation-adjusted Mueller–Hinton (CAMH) medium. The growth inhibitory activities of TST were compared with those of CLA and CFX, which are currently being using for *Mab* treatment in the clinic. As shown in Figure 2 and Table 1, TST significantly decreased the *Mab* survival rate in a concentration-dependent manner in CAMH media. TST exhibited anti-*Mab* activity against *Mab* CIP 104,536 S (smooth) morphotype and it also showed similar potent properties to the hypervirulence of a R (rough) morphotype, as shown in Table 2. In addition, TST showed a much stronger activity than that of CFX, but not CLA. To determine the TST activity against MABSC, the MIC_90_ of two *Mab* subspecies, such as *Mab bolletii* CIP108541 and *Mab massiliense* CIP108297, were evaluated in CAMH. As shown in Table 2, TST also displayed activities against *Mab bolletii* CIP108541 and *Mab massiliense* CIP108297 similar to those against *Mab* CIP 104536. These results suggested that TST is effective against all phylogenetically close *Mab* subspecies.

### 2.2. TST Exhibits Potent Activity against Mab Clinical Isolates and Drug-Resistant Strains

The fact that TST showed good inhibitory activity not only against *Mab* CIP 104536, but also against different MABSC strains raised the question of whether *Mab* clinical isolates might be susceptible to TST. In this context, we evaluated TST activity using *Mab* clinical isolates. The clinical isolates contained *Mab abscessus* and *Mab massiliense* that were confirmed by the sequencing of 16s rRNA *genes, rpoB* and *hsp65* [6]. As shown in Table 2, when *Mab* clinical isolates were cultured in the presence of various concentrations of TST, significant growth inhibition was observed. For example, the growth of *Mab* KMRC 00136-661040 R type was inhibited by 1.0 μM TST. Most *Mab* clinical isolates showed growth inhibition at much lower MIC_90_ range of 0.7–2.7 μM. These results indicated that TST is effective against clinical isolates as shown already with both *Mab* CIP 104,536 S- and R-morphotype strains. Previously, we generated spontaneously-induced drug-resistant *Mab* CIP 104,536 S strains for AMK, CFX, and CLA, namely AMK-R (MIC > 150 μg/mL), CFX-R (MIC > 120 μg/mL), and CLA-R (MIC > 20 μg/mL), at high concentrations of AMK, CFX, and CLA [25]. As seen in Table 2, TST also could inhibit the AMK-R, CFX-R, and CLA-R strains with an MIC_90_ equivalent to that seen for the wild-type strain. Thus, TST can be considered as an effective drug candidate for drug-resistant strains as well.

### 2.3. TST Regulates Proinflammatory Cytokine Production

**Figure 3:**
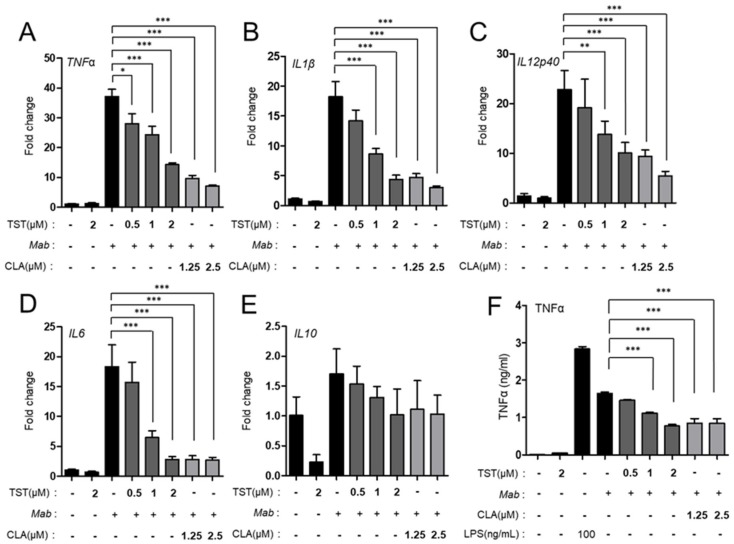
TST-mediated regulatory effects on cytokine production in bone-marrow-derived macrophage (BMDMs) during 104,536 S infection. BMDMs were infected with *Mab* CIP 104,536 S morphotype and treated with TST for 6 h; then, they were subjected to quantitative real-time Polymerase Chain Reaction (PCR) analysis for measurement of the mRNA expression of *TNF-α* (**A**), *IL-1β* (**B**), *IL-12p40* (**C**), *IL-6* (**D**), and *IL-10* (**E**), and protein expression of TNF-α (**F**). * *P* < 0.05, ** *P* < 0.01, *** *P* < 0.001, compared with *Mab*-infected cells. Data are representative of three independent experiments, and values represent mean ± standard error (SE) from three independent experiments performed in triplicate. TST, thiostrepton; CLA, clarithromycin.

**Figure 4:**
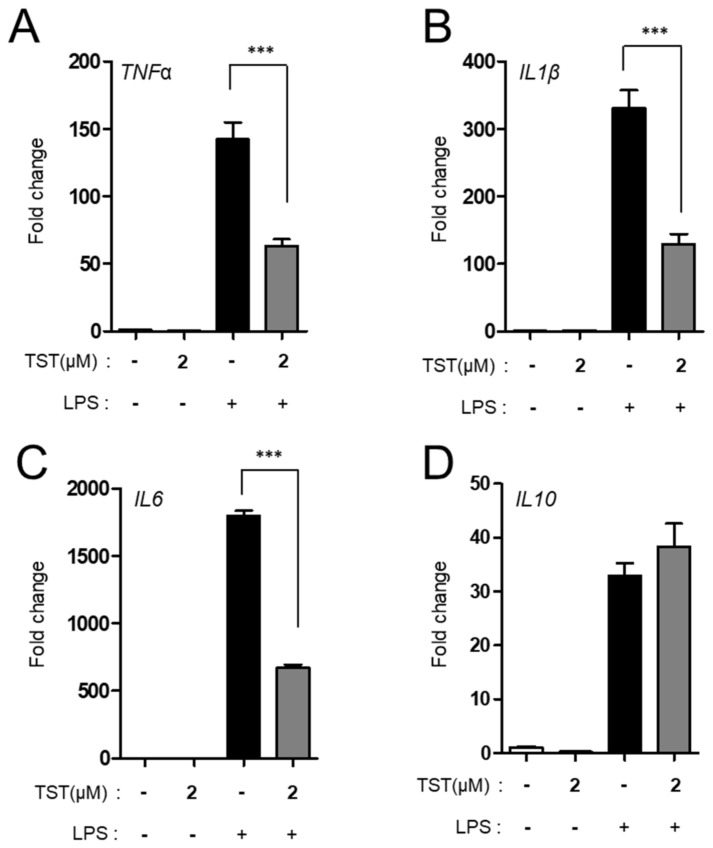
TST-mediated immunomodulatory effects on lipopolysaccharide (LPS)-induced cytokine expression in BMDMs. BMDMs were stimulated with LPS (100 ng/mL) in the presence or absence of TST (2 μM) for 6 h; then, they were subjected to quantitative real-time PCR analysis for measurement of the mRNA expression of *TNF-α* (**A**), *IL-1β* (**B**), *IL-6* (**C**), and *IL-10* (**D**). *** *P* < 0.001, compared with LPS-stimulated cells. Data are representative of three independent experiments, and values represent mean ± standard error (SE) from three independent experiments performed in triplicate. TST, thiostrepton.

When we treated *Mab* CIP 104,536 S-infected bone-marrow-derived macrophages (BMDMs) with TST, we found that mRNA expressions of proinflammatory cytokines, including tumor necrosis factor (TNF)-α, interleukin (IL)-1β, IL-6, and IL-12 p40 were significantly downregulated in BMDMs in a dose-dependent manner. However, IL-10 was not downregulated in BMDMs after *Mab* infection (Figure 3A–E). Moreover, protein expression of TNF-α was significantly decreased in *Mab*-infected BMDMs (Figure 3F). Similarly, we found that CLA treatment of *Mab*-infected BMDMs inhibited proinflammatory cytokine expression, whereas it did not affect anti-inflammatory cytokine IL-10 expression (Figure 3A–E). In addition, a positive control lipopolysaccharide (LPS) stimulation led to a significant increase in inflammatory cytokine production in the same cells. Furthermore, TST treatment significantly inhibited LPS-induced expression of several inflammatory cytokines, including TNF-α, IL-1β, and IL-6 (Figure 4A–C). However, LPS-induced IL-10 mRNA expression was not modulated by TST (Figure 4D). Thus, TST might have a dual effect on *Mab* infection via direct antimicrobial activity and regulation of host inflammation.

### 2.4. Assessment of TST Activity in Mab Infected THP-1 Cells

Similar to *M. tuberculosis*, *Mab* also infects and multiplies inside human macrophages, which shows their ability to evade antimicrobial response in macrophages [26]. Therefore, it is important to examine the ability of TST to inhibit the intracellular growth of *Mab*. The intracellular antimicrobial activity of TST against *Mab* was assessed by traditional CFU counting.

**Figure 5:**
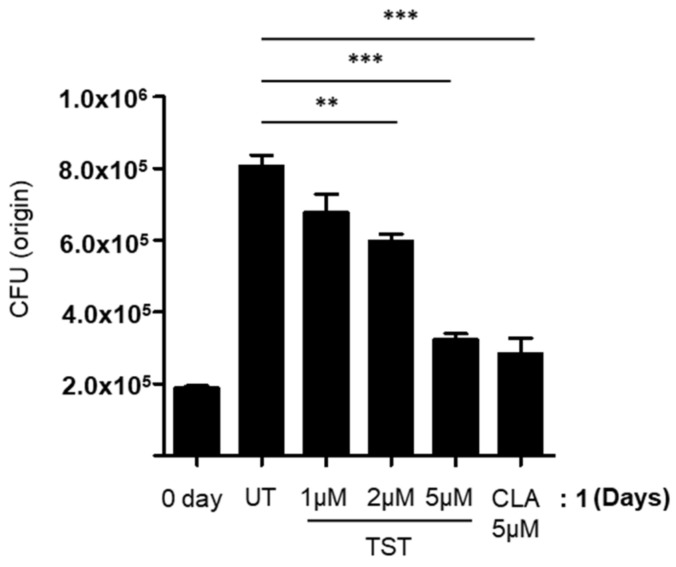
Intracellular activity of TST. The activity of TST on intracellular *Mab* CIP 104,536 S was examined in THP-1 cells. Data represent mean ± standard error (SE) from three independent experiments. ** *P* < 0.01; *** *P* < 0.001, when comparing untreated samples with samples treated with TST. UT, untreated; TST, thiostrepton; CLA, clarithromycin.

The intracellular antimicrobial activity of TST against *Mab* CIP 104,536 S was assessed after 48 h of replication inside THP-1 on a 7H10 agar plate. In this experiment, TST significantly reduced CFU of intracellular mycobacteria present at 3 days after infection at concentrations of 1, 2, and 5 μM respectively (Figure 5). This CFU reduction by TST was also comparable to that observed after CLA treatment.

### 2.5. Assessment of In Vivo TST Activity

**Figure 6:**
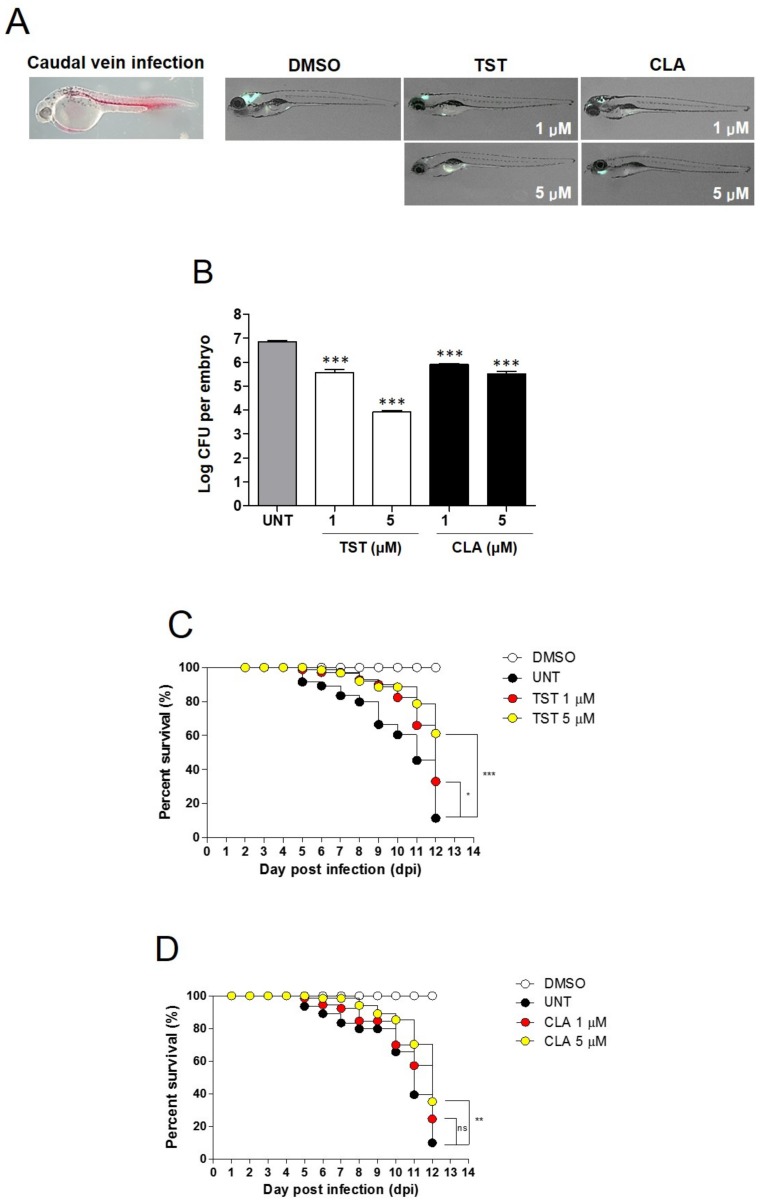
Evaluation of in vivo TST activity on *Mab* CIP 104536 R morphotype expressing mWasabi infection. (**A**) *Mab* expressing mWasabi (≈400 CFU) was infected via caudal vein infection and visualized in untreated or drug-treated embryos. (**B**) Bacterial loads of untreated, TST, and CLA treated embryos. Results are expressed as mean log_10_ CFU per embryo from three independent experiments. Significant difference (*** *P* < 0.0001) compared with untreated control. Survival of *Mab*- infected embryos treated at 1 and 5 μM of TST (**C**) and CLA (**D**) in comparison with untreated infected embryos and non-treated control (n = 20, representative of three independent experiments). Survival curves were compared with Log-rank (Mantel-Cox) test (* *P* < 0.05; ** *P* < 0.01; *** *P* < 0.001; ns, not significant). TST, thiostrepton; CLA, clarithromycin.

To test whether TST has the potential to cure *Mab* infection, we conducted a drug efficacy study by infecting zebrafish (ZF) embryo with mWasabi-expressing *Mab*, as described previously [27,28]. Since we did not have any information about TST toxicity in ZF, a wide range of TST concentrations was tested in ZF without bacterial infection to know the maximum tolerated dose (MTD). As shown in Figure S1, fish-containing water with 1 μM and 10 μM TST showed 91%–100% survival, whereas those treated with 20 μM (33.30 μg/mL) TST showed 72% survival; high doses from 40 μM to 80 μM completely reduced ZF survival after 5 days. A curved body trunk seen previously after treatment with high doses of CLA was not observed after treatment with 20 μM TST [28]. Thus, we decided to use less than 10 µM TST in ZF embryo after infection with *Mab* CIP 104536. Around 400 CFU of *Mab*-S and R morphotypes that harbored pMV262-mWasabi were injected into the caudal vein, and dissemination of infected fluorescent bacteria was observed using fluorescence microscopy after treatment with TST and CLA respectively (Figure 6A). DMSO was used as a mock control. As shown in Figure 6A, treatment of *Mab*-R-mWasabi infected ZF via caudal vein infection with 1 and 5 μM of TST showed a decrease in the degree of green fluorescence at 5 dpi. Significantly reduced mWasabi-expressing bacteria were observed when infected ZF were treated with 5 μM (8.32 μg/mL) TST, whereas untreated control fish embryo, after infection with *Mab*-R-mWasabi, showed a substantial increase in clustering of mWasabi-expressing bacteria in the head. Similar to TST, treatment with the reference compound CLA also showed a visible reduction at 5 dpi in ZF.

To determine the exact amount of TST delivered to *Mab* in ZFs, we analyzed the TST concentration in ZFs using HPLC-UV analysis. A total of 250 ZFs were treated for 5 days with TST (5 μM) and the average TST concentration in ZFs was determined. The HPLC results showed that a TST concentration of 0.25 μM/fish (0.416 μg/mL/fish) and this concentration was sufficient to inhibit the growth of *Mab* in the ZFs in vivo model system (Figure S2).

Next, *Mab* growth in the TST-treated ZF was quantified using CFU counting, which reflects the bacterial burden inside ZF after compound treatment. After 5 days of treatment with 1 and 5 μM of TST, significant bacterial reduction was observed, demonstrating that TST inhibits bacterial proliferation in infected ZF. The efficacy of TST observed at 1 μM on this CFU was comparable with that of 5 μM CLA and 5 μM TST showed significant CFU reduction in comparison with the untreated control, which showed that TST exhibits higher activity than CLA in an in vivo infection model (Figure 6B). *Mab*-S-mWasabi infected ZF also showed similar efficacy as that observed with *Mab*- R- mWasabi upon treatment with TST and CLA (Figure S3).

Next, we investigated whether increasing the TST dose could extend *Mab*-infected ZF survival. For this, the survival of ZF infected with *Mab*-R was monitored. As shown in Figure 6C, when infected ZF was exposed for 12 days to 5 μM TST, a significantly increased ZF lifespan was observed compared to that in the untreated group. Similarly, exposure to a lower dose of TST (1 μM) showed a slightly extended lifespan of infected zebrafish in comparison with untreated control. CLA also showed extended lifespan in a dose-dependent manner (Figure 6D). However, TST expanded the lifespan of ZF rather than CLA at all tested concentrations. This indicates that TST is efficient in treating *Mab* infected zebrafish. Taken together, these results suggested that TST exhibits therapeutic effects agaisnt *Mab* infections in vivo.

## 3. Discussion

An important concern today is that many studies clearly indicate that *Mab* S and R morphotypes behave differently in macrophages and animals [29,30,31]. The S morphotypes produce glycopeptidolipid (GPL) that forms the mycobacterial cell wall, and the loss of surface GPL enables the morphotype to switch from S to R. In the presence of GPL, the S morphotype is associated with lesser virulence and the R morphotype is involved in severe clinical trait and a hyper-proinflammatory response in cell and in vivo models. For example, R type *Mab* showed persistence in the lungs of infected mice and dissemination into the spleen; however, the S morphotype was cleared from the lungs within 3 weeks [32,33]. For this reason, we subjected both *Mab* CIP 104,536 S and R morphotypes to an in vitro drug-susceptibility test with TST and confirmed that TST is effective against both morphotypes. Furthermore, TST-mediated growth inhibition of R morphotype clinical isolates, such as KMRC 00136-61040 and KMRC 00200-61202, was also investigated. As shown in Table 2, TST could successively inhibit clinical isolates and MABSC growth similar to S morphotype with a narrow MIC spectrum.

This result was also confirmed in vivo using zebrafish (ZF). The ZF infection model with *M. marinum* is closely related to *M. tuberculosis* infection because it mimics early macrophage aggregation with granuloma-like lesion formation [34]. For this reason, ZF have been used to fill the gap between in vitro whole-cell drug screening and in vivo animal disease models not only for anti-tuberculosis but also for anti-*Mab* drug discovery [27,28,35,36]. In a similar manner, we injected both S and R type *Mab* into ZF and evaluated in vivo efficacy of TST using survival curve and CFU enumeration. In brief, ZF were microinjected through a caudal vein for in vivo assessment of drug efficacy against *Mab* as described previously [27,28]. In this injection system, 400 CFU *Mab* CIP 104,536 R morphotype resulted in 89% of ZF death infected at 12 dpi, while microinjection of equal numbers of *Mab* with TST treatment resulted in significantly enhanced lifespan. Furthermore, TST treatment showed excellent bacterial CFU reduction in a dose-dependent manner in comparison with CLA treatment. A similar death rate was also observed after *Mab* CIP 104,536 S morphotype injection into ZF embryos (Figure S3). Based on all the results described above, we concluded that TST exhibits anti-*Mab* capacity regardless of morphotype.

During mycobacterial infection, the inflammatory response plays a double-edged role in host- pathogen interaction. It has been reported that aberrant activation of excessive inflammatory responses exhibits a harmful host response against mycobacterial infection [37,38]. However, little is known about the regulatory effects of antimicrobial agents on inflammatory responses against *Mab* infection. TST is a protein synthesis inhibitor. However, recently, an additional mechanism of TST has been reported against *M. marinum* infection [19]. TST works through a dual mode of action targeting both parasitic bacteria and infected host cells via induction of host autophagy by promoting ER stress [19]. We found that TST treatment led to an inhibition of inflammatory responses in *M. abscessus*-infected BMDMs. Thus, we speculated that TST acts against the intracellular pathogen *Mab* by not only directly inhibiting translation of *Mab*, but also controlling the excessive inflammatory responses observed during *M. marinum* infection. Together with the previous findings indicating that TST promotes ER stress-mediated host cell autophagy [19], these data indicate that multiple action mechanisms may be implicated in TST-mediated host defense against mycobacterial infection in host cells. Further studies are required to demonstrate the potential benefits of using TST in combination with other chemotherapeutic agents.

TST has been shown to exhibit antimicrobial action in veterinary medicine and antiparasitic activity against *Plasmodium falciparum* that is responsible for malaria in humans [39]. The in vitro activity of TST against Gram-positive bacteria including *M. tuberculosis,* with novel mechanisms of action, has been highlighted as an alternative to other conventional antibiotics that are associated with drug resistance. Nevertheless, its large molecular size, poor aqueous solubility, and lack of bioavailability limits its clinical use [40]. For this reason, TST has been used only in topical ointments for treating skin infections in cats and dogs. However, given the poor pharmacokinetic profile, recently, a TST analog, LFF571, was developed by Novartis, which exhibits improved aqueous solubility and shows in vivo efficacy against *Clostridium difficile* that causes intestinal infections in humans. Currently, LFF571 has passed phase II clinical trials [41,42]. Furthermore, Wang et al., reported micelle-TST nanoparticles that improved bioavailability for inhibiting tumor growth in human xenografts [43]. These micelle-TST nanoparticles greatly enhanced its solubility and could detect tumors at 4 h and 24 h after injection, consequently reducing tumor growth rates in cancer xenografts [43]. Thus, we speculated that these newly developed versions of TST could provide a better chance to use TST in patients with *Mab* infection in the near future.

In this study, we reported the in vitro and in vivo therapeutic activity of TST against *Mab*. Furthermore, we demonstrated activity of this compound against different *Mab* morphotypes, *Mab* subspecies, a set of clinical isolates, and drug-resistant strains. We also provided evidence that TST inhibits the induction of *Mab*-induced proinflammatory cytokines in macrophages, suggesting that, in addition to its direct antimycobacterial activity, TST also alters the host immune response. The activity of TST was next addressed in infected macrophages and ZF as well. Thus, we concluded that TST is a potential anti-*Mab* candidate.

## 4. Materials and Methods

### 4.1. Ethical Statement

All ZF experiments were approved by the ethics committee concerning animal research at Gyeongsang National University (GNU-190325-E0014). Mice-related procedures were approved by the Animal Care and Use Committee of Chungnam National University.

### 4.2. Plasmid Construction and Bacterial Culture

The mWasabi (green) gene was amplified by PCR from pTEC15 (addgene #30174) using the primers mWasabi F: 5′ CGGGATCCATGGTGAGCAAGGGCGAG 3′ and mWasabi R: 5′ GGAATTCTTACTTGT ACAGCTCGTC 3′ (underlined regions indicate restriction enzyme sites), digested with BamHI and EcoRI, and inserted into the corresponding restriction site of pMV262 to yield the plasmid pMV262-mWasabi. Competent Mab cells were prepared, as previously described [44]. The pMV262-mWasabi was introduced into competent cells by electroporation; they were then recovered by shaking for 3 h at 37 °C and plated on 7H10 agar supplemented with kanamycin (50 μg/mL). Kanamycin-resistant colonies were picked and green fluorescent signals were identified using a NightSea flashlight. Mab carrying pMV262-mWasabi plasmid was used for ZF infection. Mab abscessus CIP 104,536 S- and R-type strains were kindly provided by the Laurent Kremer (Université de Montpellier, Montpellier, France). Mab bolletii CIP108541 and Mab massiliense CIP108297 were obtained from Collection de l’Institut Pasteur. Clinical isolates were purchased from the Korea Mycobacterium Resource Center (KMRC). Mab AMK-R, CFX-R, and CLA-R mutants were generated in previous work [25]. Mab strains were grown at 37 °C in cation-adjusted Mueller–Hinton medium (CAMH), and on Middlebrook 7H10 plates supplemented with 10% oleic acid-ADC (OADC).

### 4.3. MIC Determination Using Resazurin Microtiter Assay (REMA)

The MICs of the compounds were determined using the resazurin microtiter assay (REMA) as described previously [25].

### 4.4. Preparation of Human THP-1 Cell Lines for Intracellular Survival Assay

The THP-1 human monocytic cell line was purchased from American Type Culture Collective (ATCC) and cultured in Roswell Park Institue (RPMI) medium (Sigma, St. Louis, MO, USA) with 10% Fetal bovine serum (FBS-Sigma) at 37 °C with 5% CO_2_. THP-1 cells were differentiated to human macrophages by addition of PMA (Phorbol 12-myristate 13-acetate-Sigma) at a concentration of 300 nM for 6 h. Thereafter, at a multiplicity of infection (MOI) 1:3, bacteria were added and diluted with Dulbecco′s modified Eagle′s medium (DMEM) supplemented with 10% FBS. Extracellular bacteria were removed as described previously [45]. Then, the medium containing amikacin was discarded and cells were washed again three times. The cells were then treated with various concentrations of the TST. For the intracellular mycobacterial survival assay, the intracellular bacteria were extracted from THP-1 cells after 1 day, and the lysates were diluted 10-fold with PBS. Each bacterial dilution was plated onto 7H10 agar plates and incubated at 37 °C in a 5% CO_2_ incubator for at least 3 days.

### 4.5. Preparation of Bone Marrow-Derived Macrophages, RNA Extraction, Quantitative Real-Time PCR (Qpcr), and Enzyme-Linked Immunosorbent Assay (ELISA)

C57BL/6 mice were purchased from KOATECH (Gyeonggi-do, Pyeongtaek-si, Korea). Bone marrow-derived macrophages (BMDMs) were isolated from 7-week-old mice and differentiated for 4 days at 37 °C in a 5% CO_2_ incubator with medium containing macrophage colony-stimulating factor (M-CSF; JW CreaGene; Gyeonggi-do, Seongnam-si, Korea). RNA extraction and qPCR analysis were performed according to the manufacturer’s instructions. The sequences of primers are shown in Table S1. Levels of TNF-α in BMDMs were measured using ELISA kits (Cat., 558534; BD), according to the manufacturer’s instructions.

### 4.6. Microinjection of Mab into Embryos and Drug Efficacy Assessment

The rough and smooth variant strain CIP 104,536 were grown at 30 °C on Middlebrook 7H9 broth supplemented with Albumin Dextrose saline (ADS) and 0.05% Tween 80. Recombinant *Mab*-S and R morphotypes that harbor pMV262-mWasabi were maintained in logarithmic phase and were homogenized using a 26-gauge needle and sonication before being frozen and stocked by storing 5 µL aliquots at −80 °C. Prior to injection, the colony-forming unit (CFU) of the inoculum were determined by plating serial dilutions. The infectious bacteria were diluted with PBS with 0.05% Tween-20 (PBST) containing 0.05% Tween 80 and resuspended in Phenol red 0.085% to obtain around 130 CFU/nl.

Dechorionated and anesthetized zebrafish embryos at 30–48 hpf (hours post-fertilization) were injected with 3 nL of *Mab* expressing mWasabi (containing nearly 400 CFU of *Mab*) into the caudal vein using the Nanoject III Programmable Nanoliter Injector (Drummond Scientific, Broomall, PA, USA). After injection, the embryos were transferred into 96-well plates (2 embryos/well) and grown in blue fish water containing 1 g/L methylene blue at 28.5 °C to follow infection kinetics and larval survival. Two different concentrations of TST and CLA (1 µM and 5 µM) were tested by directly adding them into fish water containing the infected embryos. The infected embryos without treatment were used as a negative control. Water with the compounds was renewed once daily. The drug efficacy of each concentration was determined by observing the bacterial burden, the evolution of *Mab* within the embryos, and the kinetics of embryo survival. Dead embryos (no heartbeat) were recorded on a daily basis for 12 days to generate a survival curve. For the quantification of the bacterial load, three infected embryos (5 dpi) were collected and individually homogenized in 2% Triton X-100–PBST by using a Hand-held homogenizer (D1000; Benchmark). Several 10-fold dilutions of the suspension in PBST was plated on 7H10 containing kanamycin 50 µg/mL and BBL Mycobacteria growth indicator tubes (MGIT) PANTA (polmyxin B, amphotericin B, nalidixic acid, trimethoprim, and azlocillin; Becton Dickinson, NJ, USA) (used as recommended by the supplier) and incubated for 3 to 5 days to measure CFU.

### 4.7. TST Extraction and Quantification Using High-Performance Liquid Chromatography

A total of 250 ZFs was treated for 5 days with TST (5 μM). Before taking samples, ZFs were transferred to 2-mL microcentrifuge tubes and then washed five times with 1 mL 50% methanol in water. After washing, ZFs were homogenized using a hand-held homogenizer (D1000; Benchmark). For high-performance liquid chromatography (HPLC) quantification, a stock solution of TST was prepared in DMSO at a 1 mg/mL concentration and working standard solutions were prepared by serial dilution of the stock solution with DMSO at 1000, 500, 100, 50, and 10 μg/mL. To prepare the matrix for calibration samples, blank ZF extract was diluted 10-fold with water. Furthermore, calibration samples were prepared with the matrix at 10, 5, 1, 0.5, and 0.1 μg/mL as the final concentrations. For drug analysis, the ZF extract sample was diluted 10-fold with water. To the calibration samples and ZF extract sample (200 μL), 2 mL of acetonitrile was added, and solutions were then vortexed for 1 min. The mixture was centrifuged at 4000 rpm for 20 min. The supernatant was transferred and dried for 2 h using a centrifugal evaporator. The residue was reconstituted with 200 μL of mobile phase, vortexed for 1 min, and centrifuged at 4000 rpm for 10 min. The supernatant was transferred to HPLC vials and 50 μL was injected into the HPLC system. The HPLC system used was a Waters HPLC, consisting of an Alliance 2690 separation module, 996 PDA detector, and Empower software. The analytical column was Hydrosphere C18 (3.0 mm × 150 mm, 3 micro, YMC). The mobile phase consisted of acetonitrile, water, and trifluoroacetic acid (28:72:0.1, *v*/*v*/*v*). Separation was carried out isocratically at 40 °C, and the flow rate was set at 0.43 mL/min, with UV detection performed at 247 nm.

## Figures and Tables

**Table 1:** Drug susceptibility of *Mab abscessus* CIP 1,104,536 S morphotype.

Agent	MIC_90_ in 7H9	
	(μM)	(μg/mL)
Clarithromycin	4.2	3.1
Cefoxitin	86.9	37.1
Thiostrepton	4.1	6.8

**Table 2:** MIC_90_ of antimicrobial agents for *Mab* strains.

*Mab* Subspecies	Colony Morphology	MIC_90_ in CAMH	
		(μM)	(μg/mL)
*abscessus* CIP104536	R	0.9	1.4
*bolletii* CIP108541	S	2.2	3.6
*massiliense* CIP108297	S	1.6	2.6
*abscessus* KMRC 00136-61038	S	2.3	3.8
*abscessus* KMRC 00136-61039	S	1.2	2.0
*abscessus* KMRC 00136-61040	R	1.0	1.6
*abscessus* KMRC 00136-61041	S	1.8	2.9
*abscessus* KMRC 00200-61199	S	2.1	3.4
*abscessus* KMRC 00200-61200	S	2.7	4.5
*abscessus* KMRC 00200-61201	S	1.7	2.7
*massiliense* KMRC 00200-61202	R	2.4	4.1
*massiliense* KMRC 00200-61204	S	0.7	1.1
*M. abscessus* (CLA-R)	S	3.2	5.4
*M. abscessus* (AMK-R)	S	4.6	7.7
*M. abscessus* (CFX-R)	S	3.1	5.2

## Supplementary Materials

The following are available online, Figure S1: Maximum tolerated dose of TST in zebrafish, Figure S2: Evaluation of TST uptake level in zebrafish using HPLC-UV. Figure S3: Evaluation of in vivo TST activity on Mab CIP 104536 S morphotype expressing mWasabi infection. Table S1: The sequences of primers used for qPCR of mouse genes.
