# Supplementary material for: Thiostrepton: A Novel Therapeutic Drug Candidate for Mycobacterium abscessus Infection
Source: Molecules. 2019 Dec 10;24(24):4511. doi: 10.3390/molecules24244511 (PMC6943738; doi:10.3390/molecules24244511)
Supplement: Supplementary file 1 [file molecules-24-04511-s001.zip › molecules-602219-supplementary.pptx]

## Slide 1
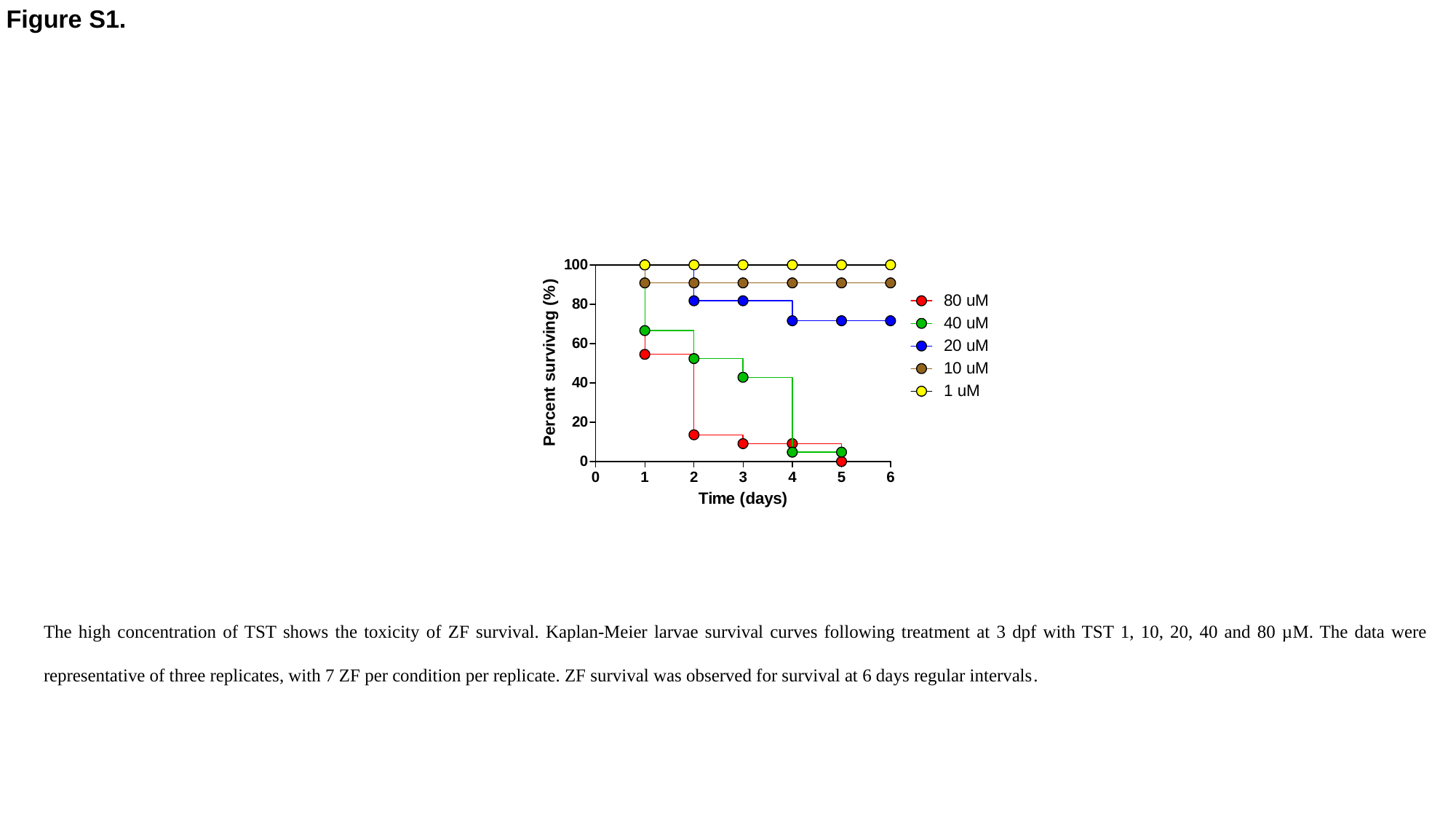

Figure S1.
The high concentration of TST shows the toxicity of ZF survival. Kaplan-Meier larvae survival curves following treatment at 3 dpf with TST 1, 10, 20, 40 and 80 µM. The data were representative of three replicates, with 7 ZF per condition per replicate. ZF survival was observed for survival at 6 days regular intervals.

## Slide 2
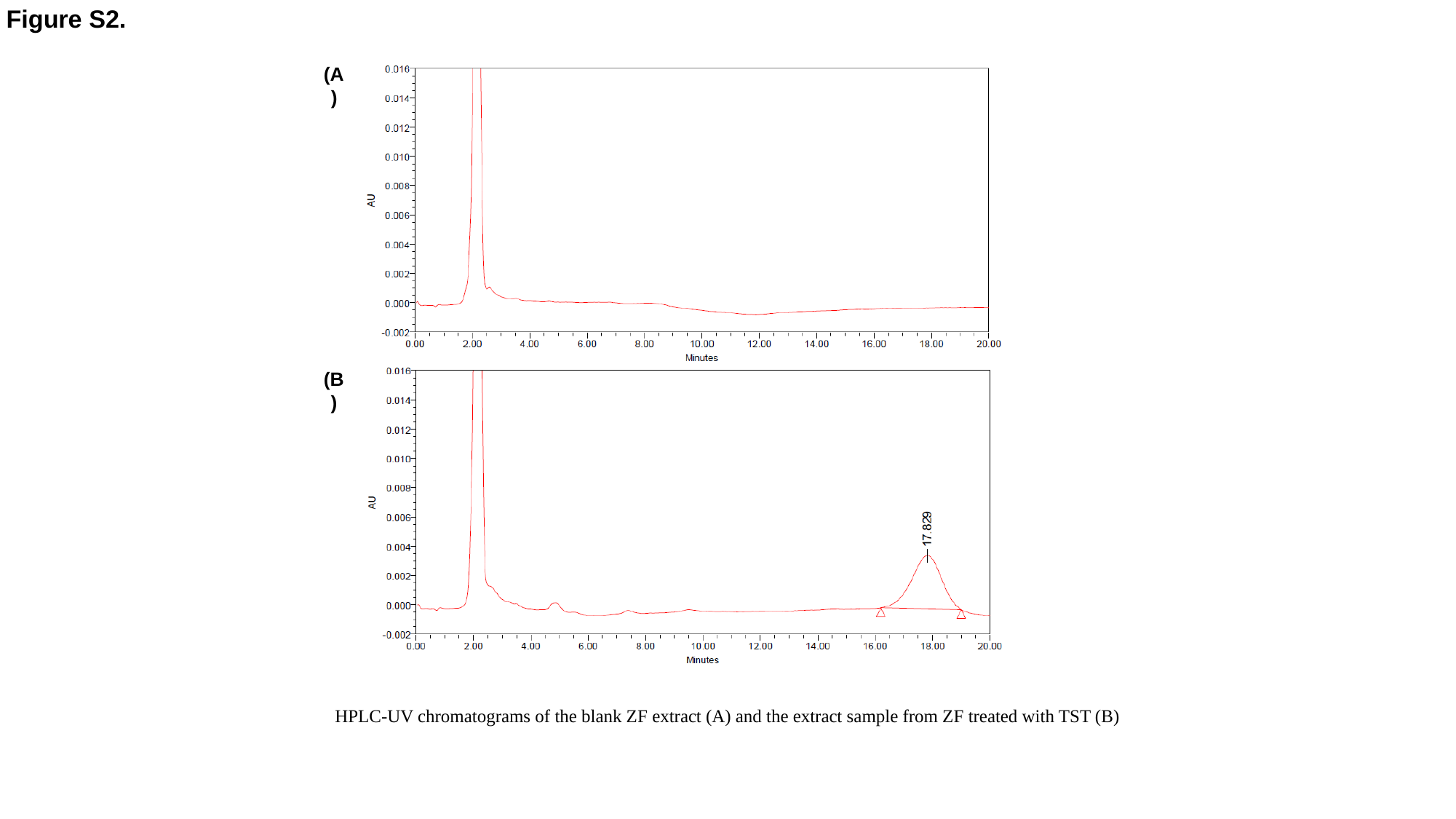

Figure S2.
(A)
(B)
HPLC-UV chromatograms of the blank ZF extract (A) and the extract sample from ZF treated with TST (B)

## Slide 3
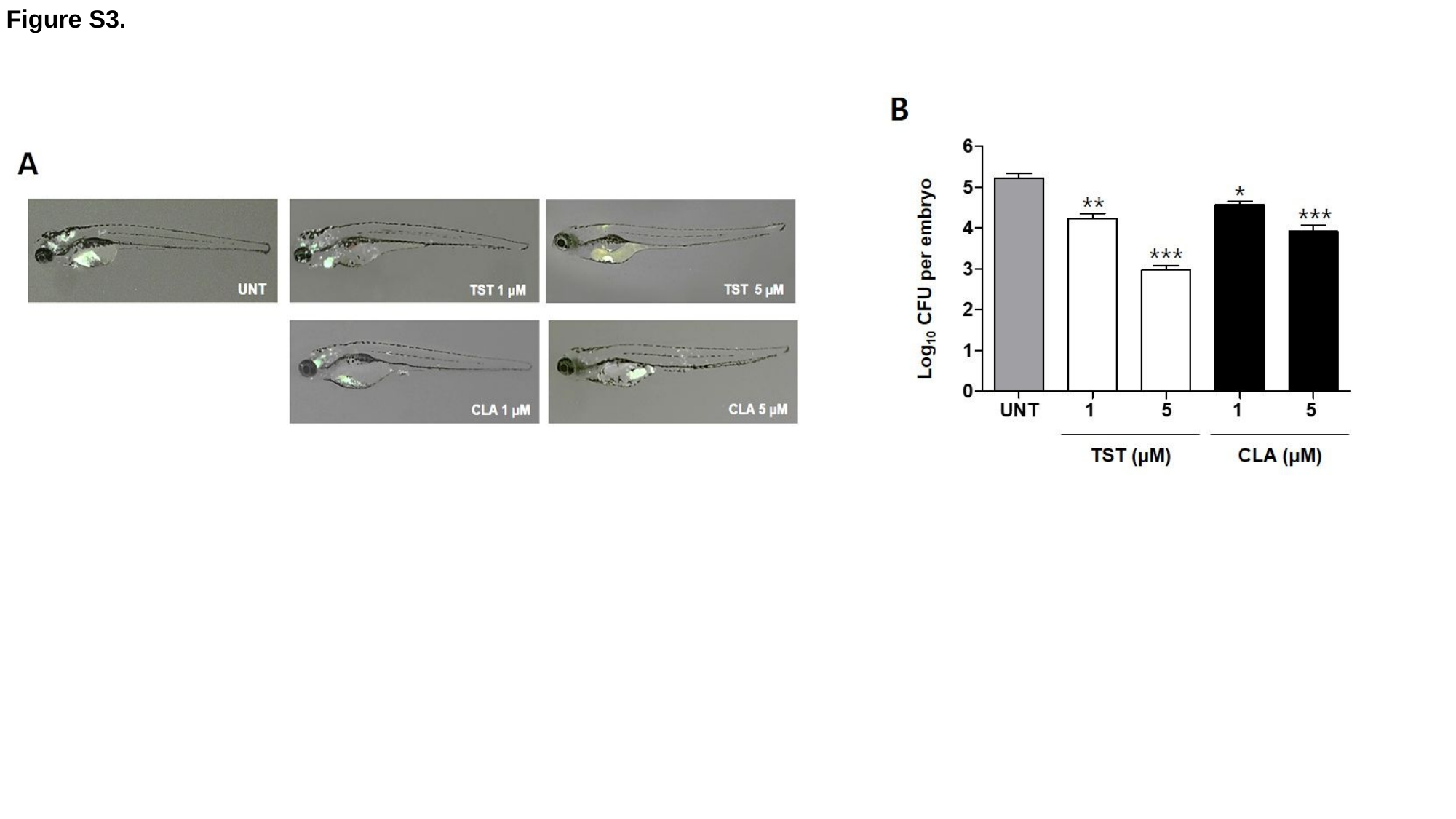

Figure S3.

## Slide 4
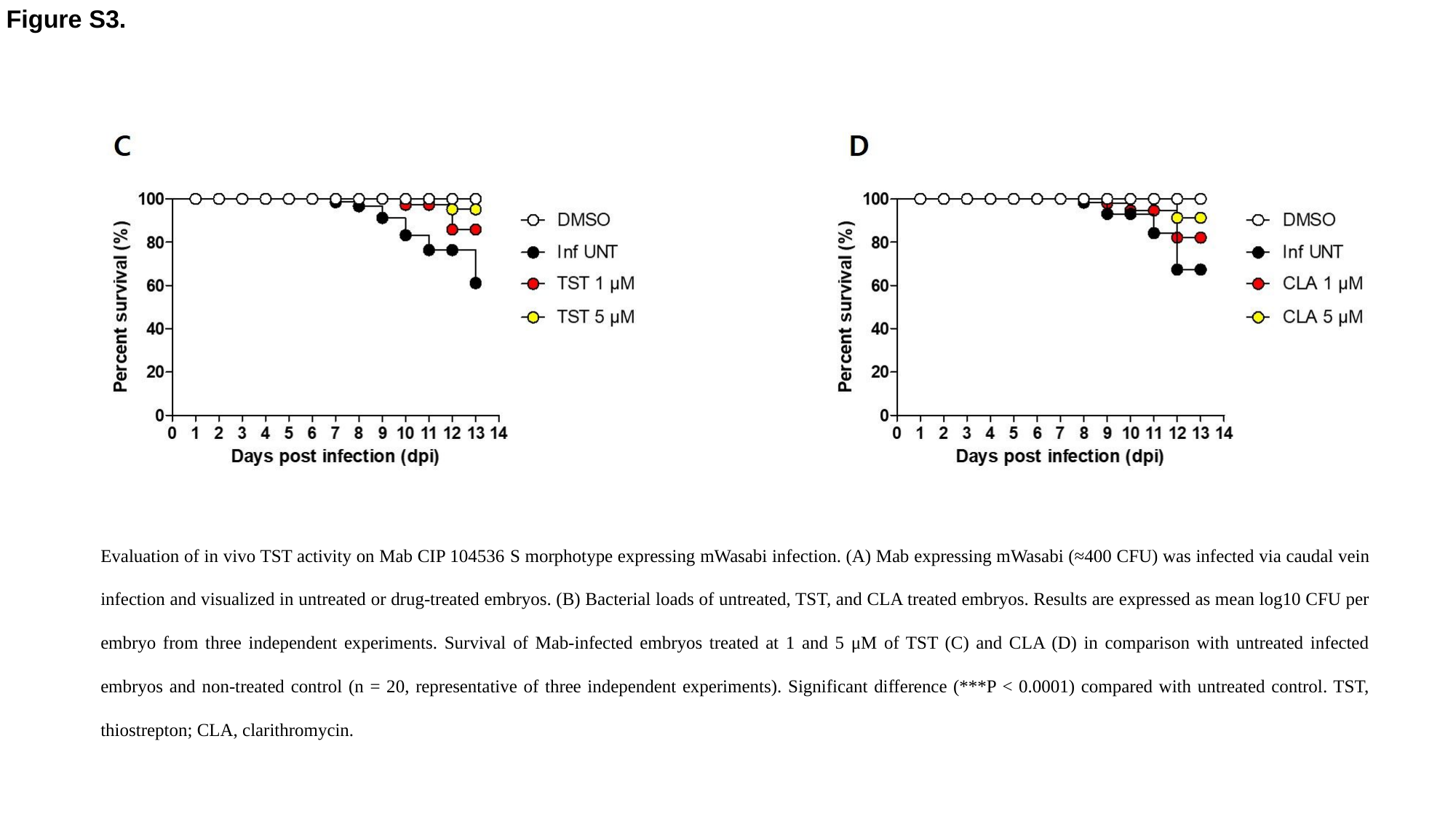

Figure S3.
Evaluation of in vivo TST activity on Mab CIP 104536 S morphotype expressing mWasabi infection. (A) Mab expressing mWasabi (≈400 CFU) was infected via caudal vein infection and visualized in untreated or drug-treated embryos. (B) Bacterial loads of untreated, TST, and CLA treated embryos. Results are expressed as mean log10 CFU per embryo from three independent experiments. Survival of Mab-infected embryos treated at 1 and 5 μM of TST (C) and CLA (D) in comparison with untreated infected embryos and non-treated control (n = 20, representative of three independent experiments). Significant difference (***P < 0.0001) compared with untreated control. TST, thiostrepton; CLA, clarithromycin.
